# Gold Nanoparticle-Based Surface-Enhanced Raman Scattering for Noninvasive Molecular Probing of Embryonic Stem Cell Differentiation

**DOI:** 10.1371/journal.pone.0022802

**Published:** 2011-08-04

**Authors:** Ramachandra Rao Sathuluri, Hiroyuki Yoshikawa, Eiichi Shimizu, Masato Saito, Eiichi Tamiya

**Affiliations:** 1 Department of Applied Physics, Graduate School of Engineering, Osaka University, Suita City, Osaka, Japan; 2 Photonics Advanced Research Center, Graduate School of Engineering, Osaka University, Suita City, Osaka, Japan; Brigham and Women's Hospital, United States of America

## Abstract

This study reports the use of gold nanoparticle-based surface-enhanced Raman scattering (SERS) for probing the differentiation of mouse embryonic stem (mES) cells, including undifferentiated single cells, embryoid bodies (EBs), and terminally differentiated cardiomyocytes. Gold nanoparticles (GNPs) were successfully delivered into all 3 mES cell differentiation stages without affecting cell viability or proliferation. Transmission electron microscopy (TEM) confirmed the localization of GNPs inside the following cell organelles: mitochondria, secondary lysosome, and endoplasmic reticulum. Using bright- and dark-field imaging, the bright scattering of GNPs and nanoaggregates in all 3 ES cell differentiation stages could be visualized. EB (an early differentiation stage) and terminally differentiated cardiomyocytes both showed SERS peaks specific to metabolic activity in the mitochondria and to protein translation (amide I, amide II, and amide III peaks). These peaks have been rarely identified in undifferentiated single ES cells. Spatiotemporal changes observed in the SERS spectra from terminally differentiated cardiomyocyte tissues revealed local and dynamic molecular interactions as well as transformations during ES cell differentiation.

## Introduction

Embryonic stem (ES) cells are pluripotent cells that have the capability to self-renew and differentiate into multiple tissue types. These cells hold great promise in the repair of damaged adult tissues by stem cell therapy, tissue engineering, and regenerative medicine [1]–[4]. *In vitro* differentiation of mouse ES (mES) cells is normally initiated by an aggregation step that leads to the formation of cell aggregates, termed EBs, which upon suitable stimulation differentiate into a wide range of specialized cell types such as neuronal cells [5], cardiac muscle cells [3], [6], and blood cells [7]. Identification of markers specific to each differentiation stage is essential for tracking the differentiation of ES cells. Techniques such as immunocytochemistry, fluorescence microscopy, polymerase chain reaction, and RNA *in situ* hybridization are generally used to measure the expression of stage-specific embryonic antigen-1, and POU family transcription factors Oct-4/Oct-3 [8], and CD9 [9] for undifferentiated state of ES cells. However, these techniques have certain limitations: they involve lengthy procedures lasting hours or days; require a large number of cells, labels, or markers; and cannot be carried out on living cells as they involve lysis, fixation, or both. Therefore, in this rapidly expanding field, the need for faster noninvasive methods to characterize and monitor the differentiation of ES cells *in situ* and in real-time is more evident than ever before.

Raman spectroscopy is a laser-based optical technique used for the analysis of molecular bonds in a sample. One advantage of Raman spectroscopy is that exogenous labeling is not required in this technique. A Raman spectrum serves as a “molecular fingerprint” of a sample, yielding information on molecular bonds, conformations, and intermolecular interactions. The approach is non-invasive and is therefore ideally suited for the study of live cells. In spite of its advantages, its practical uses have been significantly limited because the Raman scattering signal is intrinsically weaker than most other fluorescence signals. Various methods of enhancement have been developed to extend the detection limit of this method. Among these, enhancement with noble metal nanostructures, a technique termed surface-enhanced Raman scattering (SERS), was found to be particularly interesting. Using this method, it was possible to probe single molecules adsorbed onto a single silver nanoparticle [10], [11]. The sensitivity of SERS has been shown to be as high as 10^14^–10^15^, which is comparable to that of the fluorescence detection method [10], [12]. This facilitates the application of SERS to the detection of biomolecules such as DNA [13], DNA/RNA mononucleotides [14], and proteins [15]. SERS has been successfully used for labeling cells [16] and tissues [17], for multiplexed biomarker labeling to monitor apoptotic processes [18], and for real-time monitoring of single live cell signaling processes [19]. The most recent generation of SERS tags [20] can be used for the targeted detection of biomarkers such as cancer antigens found in the blood or on the cell surface. This involves the use of immunoassay approaches for *in vitro* cancer diagnosis [21], [22], *in vivo* cancer targeting and imaging [23], and mapping local pH in live cells [24] as well as in subcellular organelles in live cells [25].

For cellular and subcellular analysis with SERS, colloidal nanoparticles (such as silver or gold) are normally loaded into cells by different methods such as general incubation (fluid-phase uptake) or ultrasonication-assisted uptake [26]–[29]. Gold nanoparticles (GNPs) efficiently scatter visible light and do not blink or photobleach. Their optical properties are controlled by their plasmons, which are collective oscillations of their conduction electrons. Due to their chemical inactivity, GNPs are generally regarded to be more suitable for incorporation into living cells [26], [30], [31].

ES cell differentiation involves many biochemical and biophysical cellular changes. When cells begin to differentiate toward a specific phenotype, they produce specific proteins that assist their functions. The unique ability of ES cells to differentiate toward any phenotype indicates that there are significant biochemical differences between undifferentiated ES cells and differentiated cells. In this study, we used SERS in conjunction with GNPs to detect biochemical changes in mES cells during *in vitro* differentiation. To the best of our knowledge, there are no reports on the use of SERS to study ES cells and identify the changes that occur during differentiation.

Thus, SERS in conjunction with GNPs was used to detect biochemical differences across 3 differentiation stages, i.e., undifferentiated single ES cells, embryoid bodies (EBs), and differentiated cardiomyocytes. Cells in all 3 differentiation states were treated with GNPs by fluid-phase uptake, and nanoparticle localization inside the cytoplasm and cell organelles was studied and confirmed by transmission electron microscopy (TEM) analysis. GNP cytotoxicity on cell viability and proliferation was estimated by the neutral red uptake (NRU) assay, which is based on the uptake of the supravital dye neutral red (NR) by viable cells and MTT assay based on the mitochondrial dehydrogenase activity of metabolically active cells. The SERS fingerprints from each differentiation stage were used to distinguish the stages involved in the differentiation of undifferentiated single ES cells to terminally differentiated cardiac muscle cells *via* the EB state. Additionally, the spatiotemporal measurements of SERS fingerprints provided insight into the dynamics of molecular interactions and transformations that occur at different locations with time in differentiated cardiomyocytes.

## Materials and Methods

### ES cell culture, EB formation, and EB differentiation into cardiomyocytes

The mES cell line B6G-2 that expresses the green fluorescent protein (GFP) ubiquitously was purchased from RIKEN BRC (Tsukuba, Japan) [32]. ES cells were routinely cultured and expanded on mitotically inactivated STO cells (ECACC) [33] as a feeder layer (75 000 cells/cm^2^) in 100-mm Petri dishes (Iwaki, Japan) coated with 0.1% (w/v) gelatin (Sigma). They were maintained at 60–70% confluence to preserve an undifferentiated phenotype in humidified 5% CO_2_ at 37°C with daily medium exchange for 2 days. The cells were collected by trypsin-EDTA treatment (0.05% v/v trypsin and 0.53 mM EDTA; Invitrogen) for 2–3 min in 5% CO_2_ at 37°C. After centrifugation at 1000 rpm for 5 min, single ES cell suspensions were obtained, which were used either for subculturing or for differentiation studies. B6G2 cells express GFP ubiquitously under the β-actin promoter, and the characteristic green fluorescence from GFP helps in distinguishing ES cells from STO feeder cells.

After 2 days of mES cell culture on feeder cells, the cells were trypsinized (0.05% trypsin-EDTA) to prepare single cell suspensions, and the cell number was determined. The single cell suspension was used for EB formation by the hanging drop method [6]. EBs were formed on the inner side of the lid of a 90-mm Petri dish (Iwaki, Japan) in hanging drops that contained 800 cells in 20 µl of DMEM medium without leukemia inhibiting factor (LIF). The dishes contained 15–20 ml of sterilized Milli-Q water to prevent evaporation from the droplets. After incubation for 3 days at 37°C in 5% CO_2_, the EBs were collected and used for differentiation studies.

To induce the differentiation of EBs into cardiomyocytes, the EBs were transferred onto 0.1% (w/v) gelatin-coated 8-well glass chamber slides (Iwaki, Japan). Each well contained 2 EBs in 0.4 ml of LIF-free medium. After incubation for 12 days under the above conditions with daily medium exchange, spontaneous beatings were observed in the tissues derived from EBs.

The other cell cultivation conditions used were as follows. STO cells were inactivated by exposure to 10 µg/ml mitomycin C (Wako, Japan). The ES cell culture medium consisted of Dulbecco's modified Eagles medium (DMEM, high glucose 4.5 g/l) (Nacalai Tesque, Kyoto, Japan) supplemented with 15% (v/v) ES-cell qualified heat-inactivated fetal bovine serum (Invitrogen, USA), 2 mM L-glutamine (Invitrogen), 0.1 mM nonessential amino acids (Invitrogen), 50 U/ml penicillin, 50 µg/ml streptomycin (Invitrogen), and 0.1 mM β-mercaptoethanol (Invitrogen) in the presence of 1000 U/ml recombinant murine LIF (mLIF; Millipore, USA). mLIF addition inhibits the spontaneous differentiation of mES cells into major embryonic tissues.

### Loading of GNPs into single ES cells, EBs, and cardiomyocytes

**Figure 1:**
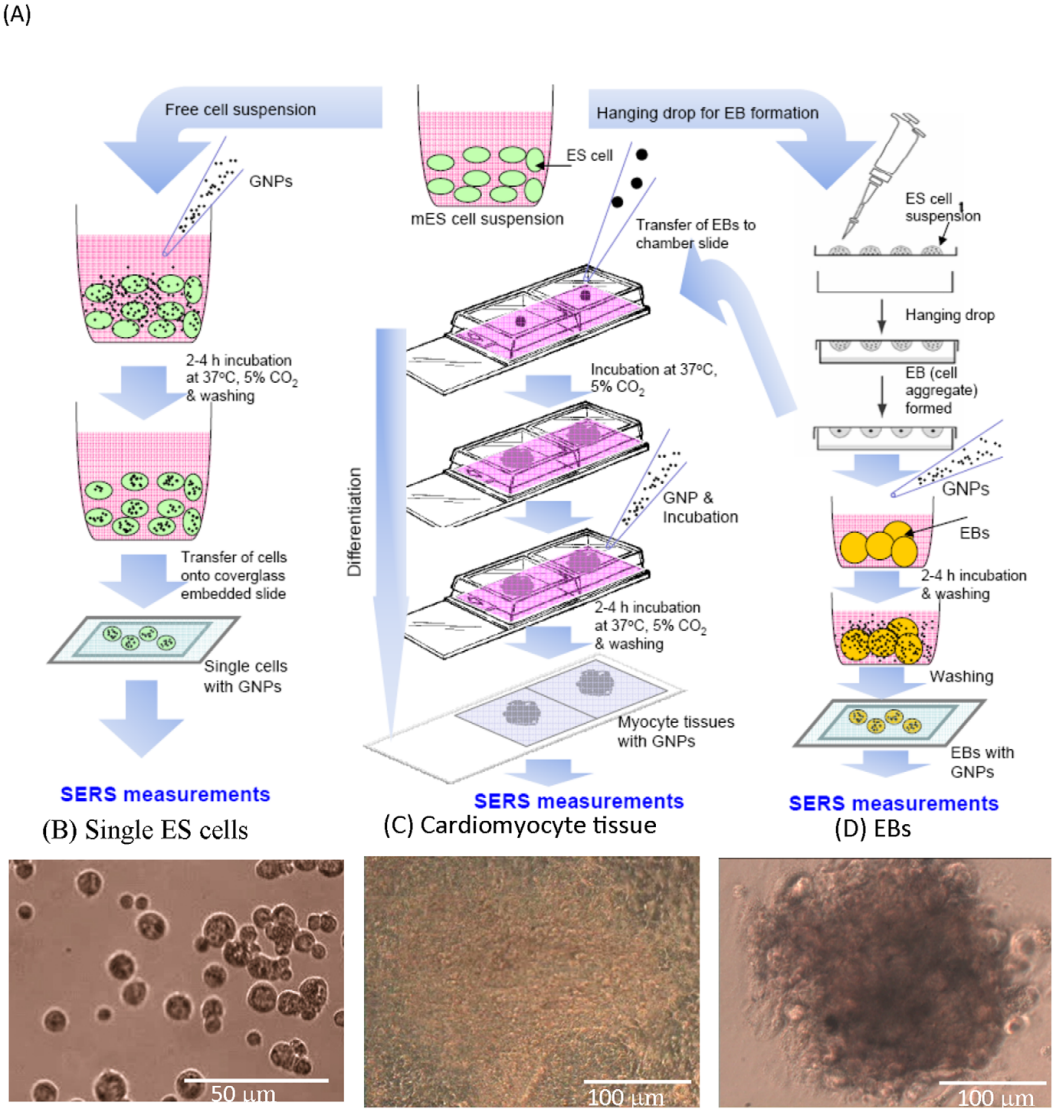
Schematic illustration showing the experimental setups and steps involved in the loading of colloidal GNP suspensions into mES cells at 3 differentiation stages, i.e., undifferentiated single cells, EBs (cell aggregates), and terminally differentiated cardiomyocytes (A). Representative optical microscopic images of undifferentiated mES cells (B), cardiomyocyte tissue (C), and EBs (D) after GNP loading.

Gold colloid suspensions in water (particle size of 40 nm, 60 nm, and 100 nm) were purchased from BBinternational Ltd. (UK). **Figure 1A** is a schematic illustration of the experimental setup used to load colloidal GNPs into pluripotent undifferentiated single ES cells, EBs, and cardiomyocytes. In the case of single ES cells, GNPs were introduced into the cells by a fluid-phase uptake method in which cells (1×10^6^/ml) were suspended in ES cell culture medium without LIF at the desired concentration (10% colloidal solution, v/v) and incubated for 2–4 h in 5% CO_2_ at 37°C. Ten- to twenty-microliter aliquots from each treatment were placed onto a cover glass embedded slide glass (Sekisui Kenkyo Plate, Japan) for microscopic observations and SERS measurements.

In the case of EBs, 40–50 EBs (200–250 µm in diameter) were taken in a 12-mm glass-bottom tissue culture dish (Iwaki, Japan) containing 800 µl ES cell culture medium without LIF. The GNP solution (10% colloidal solution, v/v) was added, and the dish was incubated for 2–4 h in 5% CO_2_ at 37°C. The EB suspension was placed on a microscopic slide where 0.3-mm silicone polymer frames served as chambers. These samples were overlaid with cover glasses and used to visualize GNPs in EBs and also to conduct SERS measurements.

In the case of cardiomyocytes, 12-d grown EB-derived cardiomyocytes in 8-well chamber slides were treated with a defined concentration (10% colloidal solution, v/v) of 100 nm GNPs and incubated for 2–4 h in 5% CO_2_ at 37°C. The chamber frame was dismantled, and a clean cover glass (0.15 mm) was placed over the chamber slide to visualize the GNPs in the tissue and to conduct SERS measurements in cardiomyocyte tissues.

Prior to the SERS measurements, all samples were washed 2–3 times with PBS to remove the culture media and GNPs present outside the cells. Optical microscopy images of the prepared samples are shown in **Figures 1B–D**.

### Evaluation of GNP cytotoxicity on cell viability and proliferation

To assess the toxicity of the gold colloids on mES cell viability, cells were treated with the desired concentration of gold colloids in water. Cell viability and proliferation were measured in a 96-well plate using the CellTiter 96 Aqueous One solution cell proliferation assay (Promega, USA), according to the manufacturer's instructions. In this assay, metabolically active cells reduce an MTS tetrazolium compound into colored and medium-soluble formazan via NADPH or NADH produced by mitochondrial dehydrogenase enzymes [34]. The formazan concentration is directly proportional to the number of viable cells and is estimated from the absorbance at 490 nm using a 96-well microplate reader (SH-1000, Corona Electronics, Ibaraki, Japan).

Mouse ES cell suspensions of varying cell densities (1.2×10^5^, 1.8×10^5^, and 2.4×10^5^/ml) in DMEM culture medium were treated with 10% (v/v) GNP suspensions (40, 60, and 100 nm) in different vials and incubated for 1 h in 5% CO_2_ at 37°C. One hundred microliters of the sample mixture containing 12 000, 18 000, or 24 000 cells and nanoparticles was transferred to each well of a 96-well plate and incubated for another 2 h and 24 h. At the end of the incubation period, 20 µl of MTS reagent (Promega, USA) was added to each well, and formazan formation by metabolically active cells was measured every hour for 4 h by continuing the incubation under the above conditions. Another set of experiments were performed to determine the dose-dependent toxicity of GNPs. Different concentrations (0, 5, 10, 15, and 20%) of GNPs (40 and 100 nm) were loaded into wells containing 24 000 ES cells. After 72 h of incubation, MTS reagent (20 µl) was added to each well. The formazan absorbance was estimated for 4 h, and the result corresponds to the ES cell proliferation in the presence of GNPs. Cells without nanoparticles served as controls, while medium and medium with nanoparticles served as blanks in the experiment. The absorbance derived from the experimental samples was subtracted from the respective blanks to obtain the net absorbance for each treatment. All the experiments were performed in triplicates and repeated twice.

### NRU assay for determining GNP cytotoxicity effects on ES cells

The NRU cytotoxicity assay is a cell survival/viability assay based on the ability of viable cells to incorporate and bind NR, which is a weak cationic supravital dye that readily penetrates cell membranes by nonionic diffusion and predominately accumulates intracellularly in lysosomes [35]. Alterations of the cell surface or the sensitive lysosomal membrane lead to lysosomal fragility and other changes that gradually become irreversible. Such changes produced by toxic substances or nanoparticles can lead to decreased NR uptake and binding, thereby allowing the spectrophotometric differentiation of viable, damaged, or dead cells. Cytotoxicity is expressed as the concentration-dependent reduction in NR uptake after chemical exposure, thus providing a sensitive integrated signal of both cell integrity and growth inhibition.

ES cells at different cell densities (0.6×10^5^, 0.9×10^5^, and 1.2×10^5^ cells/ml in DMEM culture medium) were treated with GNPs of various sizes (40, 60, and 100 nm) to a final concentration of 10% (v/v) colloids, which were prepared in separate vials. Next, 200 µl each of sample mixture containing 12 000, 18 000, or 24 000 cells and nanoparticles was transferred to each well of a 96-well plate and incubated for 24 h at 5% CO_2_ and 37°C. At the end of the incubation period, the medium was aseptically aspirated from the wells, and 100 µl of NR working solution (40 µg/ml, 1∶100 NR stock solution with culture medium) was added to each well. The plate was left for incubation under the above conditions for 2 h. The NR stock solution (4 mg/ml) was prepared by dissolving 40 mg of NR in 10 ml of PBS. The NR staining solution was removed from each well by aspiration. The cells were then washed with 150 µl of PBS, and the fluid was removed by aspiration. This was followed by the addition of 150 µl of NR destaining solution (50% ethanol, 49% deionized water, and 1% glacial acetic acid, equivalent to 10 ml water, 10 ml ethanol, and 0.2 ml glacial acetic acid), and the plate was rapidly shaken on a microtiter plate shaker for 10 min or until the NR dye had been extracted from the cells and had formed a homogeneous solution. The absorbance of the NR extract was measured at 540 nm in a microtiter plate reader spectrophotometer (SH-1000, Corona Electronics, Ibaraki, Japan) using blanks that contained no cells as references. All the experiments were performed in triplicates and repeated twice.

### TEM analysis

GNP uptake was examined by TEM to confirm that the GNPs had actually entered the cells and were not just attached to the cell surface. For TEM imaging, cardiomyocyte tissue samples treated with GNPs were fixed in 2.5% (v/v) glutaraldehyde (TAAB Laboratories, UK) dissolved in 0.1 M sodium phosphate buffer (pH 7.0) (Wako, Japan), and the samples were incubated for 30 min at room temperature. The samples were then washed 3 times with 0.1 M phosphate buffer containing 8% sucrose, followed by post fixation for 45 min at room temperature with 2% (w/v) osmium tetroxide (EM grade) in sucrose-phosphate buffer. This was followed by washing with sucrose-phosphate buffer and sequential complete dehydration of the specimen in a series of increasing ethanol concentrations (60 to 100%). The samples were then infiltrated and embedded in epoxy resin. Ultrathin sections (80 nm), which were taken parallel to the bottom of the culture dish, were placed on TEM copper grids and poststained with 2% aqueous uranyl acetate for 20 min, followed by staining with lead citrate for 4 min at room temperature [36]. The grids were examined, and TEM images were acquired with iTEM (SIS, Germany) software at an accelerating voltage of 80 kV using a Jeol 1200EX transmission electron microscope (Jeol Ltd., Japan) equipped with a Morada digital CCD camera system (Olympus SIS, Germany).

### Experimental setup for imaging and SERS measurements

**Figure 2:**
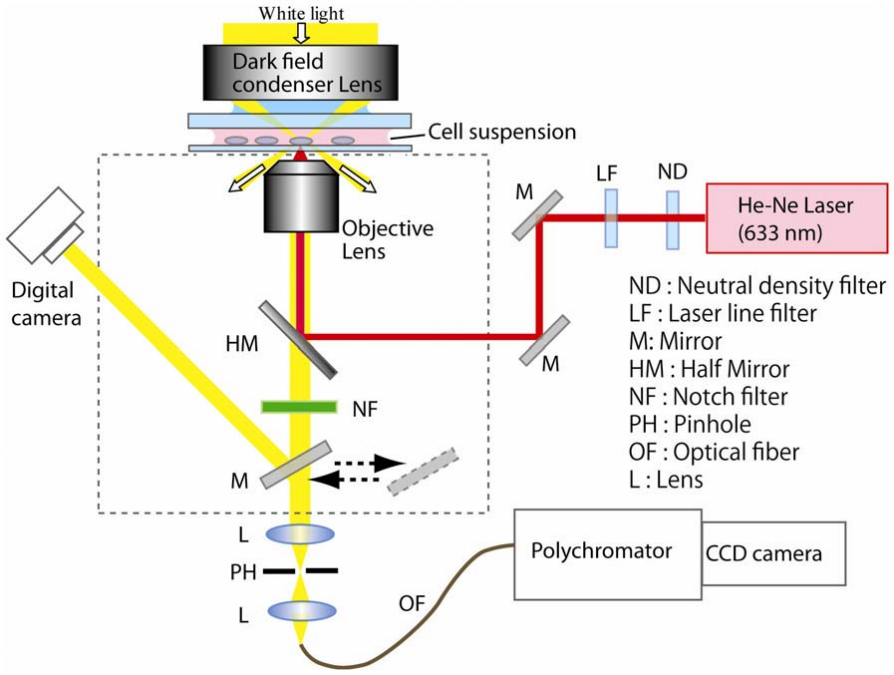
Illustration of the experimental setup employed for SERS spectra measurements. He-Ne laser excitation (632.8 nm) was delivered to a sample placed on an inverted microscope, and this sample was used for SERS spectral measurements from single ES cells, EBs, and cardiomyocyte tissue samples. The SERS spectra were acquired by detection of scattering signals sent through a pinhole and delivered into the polychromator and CCD camera.

The experimental setup employed for measuring SERS signals from all 3 ES cell differentiation stages, i.e., single cells, EBs, and cardiomyocytes, is shown in **Figure 2**. From each stage, samples loaded with GNPs were placed onto microwell glass plates (Sekisui Kenkyo Plate, Japan). The samples were then positioned on the sample stage of a Zeiss inverted optical microscope (Axiovert 200). White light from a tungsten-halogen lamp illuminated the samples through a condenser lens, and dark and bright-field images were taken with a digital camera (Olympus, SP-510UZ). The Raman excitation source was a 632.8 nm He-Ne laser beam (Melles Griot, 05-LHP-151) that was focused on the sample through an oil immersion objective lens (×100 magnification). The size of the laser focus was approximately 1 µm in diameter in the focal plane, and the power of the laser beam through the objective lens was 130 µW. Raman scattering light from the sample was introduced into a spectrometer consisting of a polychromator (Acton Research, Spectra Pro 300i) and a CCD camera (Roper Scientific, PI-MAX1024HG18). A notch filter (Semlock, NF01-488/532/635) placed in the optical path of the signal light cut the excitation laser beam. Raman signals were collected in the spectral interval of 400–2000 cm^−1^.

## Results and Discussion

### Ultramicroscopic imaging of GNP accumulation

**Figure 3:**
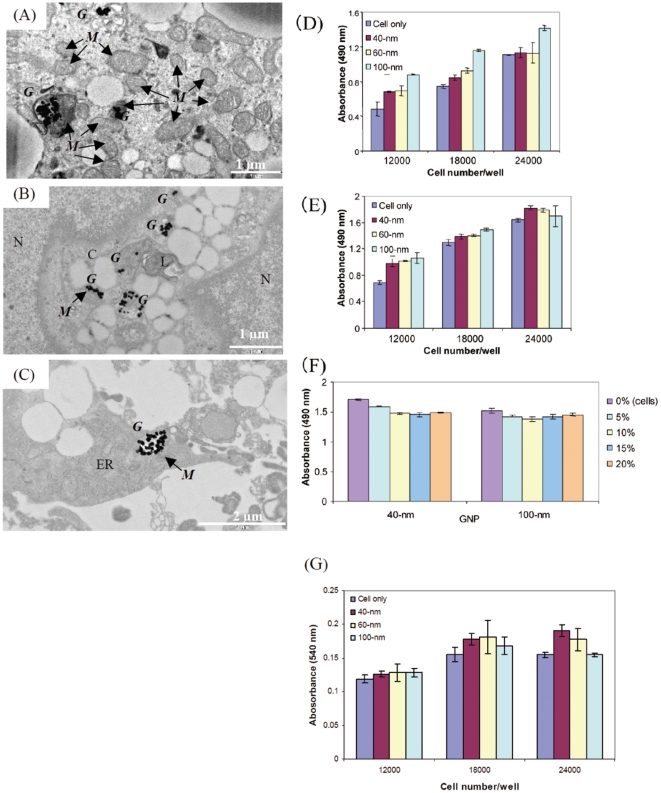
TEM imaging and GNP cytotoxic effects on ES cell viability and proliferation. TEM images showing GNP localization in ES cells, EBs, and cardiomyocyte organelles (A–C). It was observed that 40-nm GNPs accumulated in the mitochondria, secondary lysosome, and other cytoplasmic organelles (A), 60-nm particles localized in the mitochondria and secondary lysosome (B), and 100-nm particles accumulated in the mitochondria and endoplasmic reticulum in the cytoplasm (C). G, M, L, N, and C indicate GNP, mitochondria, secondary lysosome, nucleus, and cytoplasm, respectively. ER indicates the endoplasmic reticulum, and small dark spheres represent ribosomes attached to the ER. TEM images captured at a voltage of 80 kV with a Jeol 1200EX electron microscope. Cytotoxic effects of GNPs (40, 60, and 100 nm) on ES cell viability for 2 h (D) and on cell proliferation for 24 h (E). The effect of the GNP concentration on ES cell proliferation for 72 h (F) at varying ES cell densities. The experiments were performed in a 96-well plate using the MTS assay. **Figure 3D and 3E** show the values at the end of 3 h of incubation with the MTS reagent, while **Figure 3F** shows the results after 4 h of incubation. The cytotoxic effects of GNPs on ES cell viability and proliferation after 24 h (G) at varying cell densities. The experiments were performed in a 96-well plate using the NRU assay.

Nanoparticle entry and localization inside ES cells, EBs, and cardiomyocyte tissues were confirmed by TEM. The detailed TEM procedure is described in the supporting information. The results of the TEM observations are presented in **Figures 3A–C**. The TEM images showed that GNPs were taken up by ES cells and were localized in the cytoplasm and certain cell organelles, mostly in the form of aggregates. It should be noted that aggregated nanoparticles ordinarily have high SERS activity [37]. GNPs were localized in the perinuclear region and in cell organelles such as the mitochondria, secondary lysosomes, and rough endoplasmic reticulum but not in the nucleus (**Figures 3A–C**). It is very interesting to note that GNPs, independent of size, accumulated in the mitochondria, which is regarded as the cell's “machinery of life.” GNPs were internalized in the mitochondria and did not cause apoptosis or necrosis of the cells (**Figures 3A–C**). The mitochondrial cristae were clearly visible in the electron micrographs of the cells (**Figures 3A, B**). The TEM images revealed that most GNPs were embedded in the ES cells in the form of aggregates. Induction of GNP aggregation by the culture medium was also verified by incubating the same concentration of GNPs in the ES cell culture medium for the same time and then examining the samples by dynamic light scattering. The results showed that the particle size increase was less than 30%. The formation of large aggregates was not observed in the TEM images. This suggests that the localization and aggregation of GNPs occurs after GNP uptake by cells. Nanoparticles that had accumulated in different cell organelles appeared as aggregates; this phenomenon has also been reported by Kneipp et al. [26]. For the GNP uptake measurements, 80–100 cells were analyzed for GNP of each size. In the majority of the cells, GNPs were found to be localized in the mitochondria (data not shown). In addition, GNP aggregates were found in the secondary lysosome and other cell organelles. Generally, it is difficult to direct nanomaterials to a specific cell organelle such as the nucleus or mitochondria, and directed entries are commonly mediated by specific signal peptide sequences [38]–[43]. In the present study, we found that clustered GNPs were present in the mitochondria of ES cells without such surface modifications. This could be a very useful feature in future studies. It is clear from the TEM images that GNP treatment did not result in any cytotoxic effects on the cells, such as apoptosis or necrosis. We further confirmed GNP accumulation inside the cells by using energy-dispersive X-ray spectroscopy (EDX) with the Horiba EMAX-7000 system (**Figure S1**).

### GNP cytotoxicity on ES cell viability and proliferation

The TEM imaging results suggested that ES cells loaded with GNPs were healthy and had normal ultrastructural features that were similar to those of control cells that did not contain nanoparticles. We further studied GNP cytotoxicity by exposing ES cells to GNPs over a period of time and then measuring ES cell viability and proliferation by the MTS and NRU assays. The cell viability and proliferation were unaffected both in short-term (2 h) and long-term (24 h) incubations at all the cell densities tested and with GNPs of all 3 sizes (**Figures 3D, E**). Instead, ES cell growth and proliferation increased in the presence of GNPs. The increase was greater at all cell densities that contained 100 nm GNPs in comparison to controls without GNPs and cells with 40 and 60 nm GNPs subjected to short-term (2 h) and long-term (24 h) exposure (**Figures 3D, E**). On the other hand, the results from cells exposed to different concentrations (5, 10, 15, and 20%, v/v) of GNPs (40 and 100 nm) for 72 h showed that in comparison to control cells, i.e., cells without any GNPs, cell growth and proliferation were unaffected at all the GNP concentrations tested. These results suggested that higher GNP concentrations (20%) did not affect ES cell proliferation even after longer exposure times (**Figure 3F**). The GNP cytotoxicity effects on ES cells were confirmed by using the NR uptake assay, which tests the ability of viable cells to incorporate the NR dye into lysosomes. Both cell viability and proliferation were uninhibited after 24 h incubation at all cell densities tested and with GNPs of all 3 sizes (**Figure 3G**); instead, the presence of GNPs tended to increase ES cell growth and subsequent proliferation. This increase was prominent at higher cell densities treated with 40 and 60 nm GNPs (**Figure 3G**). Microscopic observations also confirmed that GNP-loaded cells showed higher growth. This was evidenced from the fact that the number of NR dye uptake cells was higher in these wells than in control wells that did not contain GNPs (not shown). These results correlated well with those of the MTS assay, which were based on the reduction of the MTS tetrazolium compound by the mitochondrial dehydrogenase enzymes of metabolically active cells. We also confirmed the cytotoxic effects of GNPs by using Cayman's lactate dehydrogenase (LDH) cytotoxicity assay kit. In this assay, cell death is measured in response to chemical compounds or environmental factors through a 2-step reaction. Moreover, the assay measures the membrane integrity, which is an indicator of the effect of GNPs on cell viability and proliferation. The results showed that in comparison to control cells, the presence of GNPs in cells protected them from regular damage or lysis. This was demonstrated by the lower release of LDH from cell membranes into the culture medium, leading to a decrease in the reduction of tetrazolium salt (INT) to highly colored formazan that absorbs at 490 nm (data not shown). The results from these 3 assays confirmed that the GNPs tested (40, 60, and 100 nm) supported the growth and proliferation of mES cells. Previous reports on the cytotoxic effects of GNPs on human leukemia (K562) cells suggested that although GNPs were not acutely toxic, a gold-salt (AuCl_4_) precursor solution showed greater than 90% toxicity at 200 µM [30]. In the present study, the presence of GNPs stimulated ES cell growth and proliferation, suggesting that the free radicals formed during cellular growth were probably quenched by GNPs (**Figures 3D, E, G**). In fact, it is well-known that free radical formation during cellular growth hinders cell proliferation [44] and that GNPs act as antioxidants to overcome such problems [31], [45]–[47]. A recent study in which a human prostate cancer cell line (PC-3) was treated with 30–90 nm GNPs concluded that no toxic effect could be attributed to the ability of GNPs to reduce the amount of potentially harmful reactive oxygen species (ROS) in cells [48]. It has been reported that small GNPs (0.8–15 nm) showed significant size-dependent toxicity in fibroblasts, epithelial cells, and melanoma cells [49]. However, in the present study, no cytotoxic effects were observed on cell growth and cell proliferation (**Figure 3F**). In comparison to GNP, fluorescent polymer nanoparticles reduced the viability of mES cells by 40% [50]. These results suggest that ES cell viability and proliferation were not inhibited by the GNPs present. Instead, GNPs appeared to inhibit ROS generation, resulting in increased viability and proliferation of ES cells.

### Imaging of ES cells, EBs, and cardiomyocytes with GNPs followed by SERS measurements

**Figure 4:**
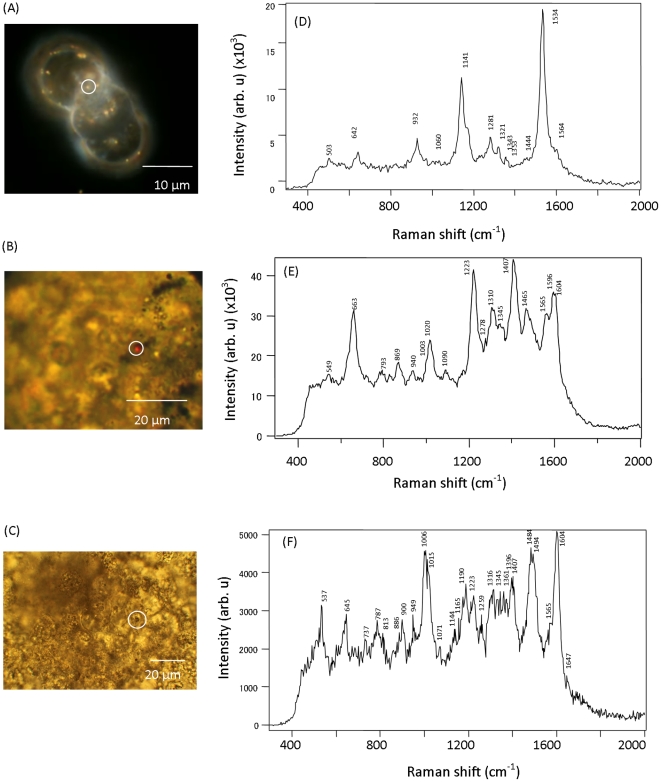
SERS spectra characteristic of the 3 differentiation stages of mES cells. The dark-field microscopic image of undifferentiated single ES cells loaded with 100 nm GNPs (A), and the corresponding SERS spectrum from the particle encircled inside the ES cell (D). The bright-field microscopic image of EBs treated with 100 nm GNPs and gold nanoaggregates visible in the dark (B). The corresponding SERS spectrum measured from the encircled area of EB (E). The bright-field microscopic image of 12-d-grown cardiomyocyte tissues loaded with 100 nm GNPs appeared as aggregates in the circled area (C) and in the corresponding SERS spectrum (F). Images captured by a digital camera (Canon, Japan) after excitation with a He-Ne laser (632.8 nm) at a power of 130 µW using a ×100 objective lens. SERS spectra of both ES cells and EBs acquired with 30-s laser acquisitions. The SERS spectrum of the cardiomyocyte tissue samples was acquired with 1-s acquisitions.

For the SERS measurements, single ES cells, EBs, and cardiomyocyte tissues loaded with GNPs were observed under an optical microscope using a ×100 objective lens. Microscopic images of representative samples are shown in **Figures 4 A–C**. The images in **Figures 4B and 4C** are unclear because EBs and cardiomyocytes are too large and bulky for microscopic imaging with a high magnification lens. Only some parts of the EB and cardiomyocyte are in focus, and overlap of the defocused region degrades the image quality. We could confirm a difference in nanoparticle aggregation in single ES cells in comparison to EBs and terminally differentiated cardiomyocyte tissues. The GNPs in EBs and cardiomyocytes were self-assembled into submicron-sized aggregates that could be observed by bright-field imaging (**Figures 4B and 4C**). Since GNPs and nanoaggregates in single ES cells are too small to be observed by bright-field imaging, dark-field imaging was employed to detect these, as shown in **Figure 4A**. The incubation time and culture medium were the same in all nanoparticle uptake studies; therefore, the difference in the size of aggregates formed could be attributed to differences in the cell function state in EBs and cardiomyocytes.

SERS measurements were performed by focusing a He-Ne laser beam onto these GNPs. The position of the laser focus was adjusted on a GNP or GNP nanoaggregate by moving the sample stage. Adjustment of the laser focus was confirmed by backscattering of the light of the He-Ne laser beam due to strong Rayleigh scattering by GNPs, as shown in **Figure 4B** (red spot in a white circle). Representative SERS spectra measured from undifferentiated single cells, EBs, and cardiomyocytes loaded with 100 nm GNPs are shown in **Figures 4D–F**. Apart from specific differences in the Raman peaks (discussed below), the spectra from the EBs and cardiomyocyte tissue samples showed noticeable differences from that of single ES cells. First, more SERS peaks were collected for the EBs and cardiomyocyte tissue samples. Second, the SERS spectra acquired from the EBs and cardiomyocytes showed background at the base level, unlike the spectra from single ES cells, which may be due to the overlap of multiple Raman peaks (**Figures 4D–F**).

Using the same laser excitation, we could not collect any Raman spectral data from a control cell without GNPs. Only spots with GNPs yielded an SERS spectrum. However, half of the measured GNPs did not yield an SERS spectrum. This is expected because the SERS spectrum arises from molecules that are adsorbed at a specific site of GNPs where the electromagnetic field is enhanced. In addition, the enhancement factor of the electromagnetic field is strongly dependent on the nanoparticle shape and aggregate structure [11], [26]. Thus, there is low probability that SERS-active GNP is spontaneously formed in the cell. However, we succeeded in measuring clear Raman peaks from ∼10% of these nanoparticles.

As reported earlier by some groups, the SERS spectra from gold or silver nanoparticles in biological cells consist of many complicated vibration bands, and the spectral profile differs from particle to particle. This spectral variation is caused by the presence of various macromolecules in the cells, such as proteins, lipids, nucleic acids, and carbohydrates. In many cases, the assignment of Raman peaks to specific vibrational modes of a biomolecule is considerably difficult. This is a common issue in Raman analysis of biological cells. Therefore, our band assignment, which is summarized in **Table 1** and discussed below, is tentative and is based on data reported in literature [51]–[63].

**Table 1:** Tentative assignment of SERS peaks derived from undifferentiated ES cells, EBs, and differentiated cardiomyocyte tissues [51]–[63].

ES cell (cm^−1^)	Peak assignment	Embryoid body (cm^−1^)	Peak assignment	Cardiomyocyte tissue (cm^−1^)	Peak assignment
642	C-C twist tyrosineO-P-O stretch in DNA	663	T,G (DNA bases)C: ring breathing	645	C-C twist tyrosineC-N stretch in lipid/adenine
787	O-P-O stretch in RNAHistidine	793	C-DNA: phosphodiester stretchingv(C-C), α-helix	737	O-P-O stretch in DNAO-P-O stretch in RNA
813	Proline ring v(C-C)PO_2_^−^ str (protein)	869	Phenylalanine ring vibrationDNA backbone: C-O stretch	787	C-O-C skeletal mode; disaccharideC-C skeletal stretch in protein
918	Ribose phosphate		Histidine phosphate	813	P:C-C skeletal mode (random)
932	P: Amide III (O-sheet)P: Amide III	9401000–1003	Ribose phosphateP: Amide III(O-sheet)	886	PhenylalaninePO_2_^−^
1060	C: base (cytosine)G (guanine), CH def	1020	P: Amide IIIC: base (cytosine)		Ribose phosphateC-C stretching in protein
1141–1146	A: baseProteins: CH_3_ deformation		A: ring Proteins: γ_T_ (CH_2_-CH_3_)A: base	900	Nucleotides: base & Try, PheP: Amide III (O-sheet)
1220	Alanine, Try, G: baseδCH_2_	1090–1096	Proteins: CH_3_ deformationProteins: COO- symmetric stretching	949	P: Amide IIIC: base (cytosine)
1270–1289	A, G, TP: Amide IIC = C (lipid);	1138–11411223	A, G, TP: Amide IIC = C (lipid);	1006	Proteins: γ_T_ (CH_2_-CH_3_)A: baseProteins: CH_3_ deformation
1321	A, C, G	1275–1283	A, C, GMitochondria	10711144	Alanine, Try, G: baseProteins: COO- symmetric stretching
1335					A, G, T
				1165	P: Amide II
		1310			C = C (lipid);
1353–1367				1190–1196	A, C, G
					Mitochondria
1444		1337			P: Amide I
1484				1223	
1534–1567					
		1394–1415		1256–1275	
				1316–1318	
		1481–1502			
		1520–1565		1329–1340	
		1596–1604		1361	
				1404–1407	
				1484–1502	
				1531–1567	
				1596–1604	
				1647	

### SERS profiles from undifferentiated single ES cells

In **Figure 4D**, the Raman peak at 642 cm^−1^ originated from the C-C twist of tyrosine, and the peak at 932 cm^−1^ corresponded to the proline ring *v*(C-C) vibration mode [51]–[53]. The strong peak at 1141 cm^−1^ corresponded to the ribose phosphate of nucleotides, and peaks 1281, 1321, and 1343 cm^−1^ were assigned to the cytosine, guanine, and adenine bases, respectively [53], [54]. A very strong peak at 1534 cm^−1^ was ascribed to lipid stretches in single ES cells [51].

### SERS profiles from EBs

The SERS spectra derived from EBs and terminally differentiated cardiomyocytes showed many peaks, unlike the spectra from undifferentiated mES cells (**Figures 4E and 4F**). In **Figure 4E**, the peak at 663 cm^−1^ was assigned to thymine and guanine bases. The very strong peak at 1223 cm^−1^ was characteristic of protein amide III (O-sheet) [57] while that at 1310 cm^−1^ originated from the adenine ring as well as the γ_T_ (CH_2_-CH_3_) twisting of proteins [53]. The strong peak at ∼1400 cm^−1^ was assigned to the COO- symmetric stretching of amino acids [58], and the weak peaks at 1565 cm^−1^ and 1580 cm^−1^ were ascribed to amide II, guanine and adenine bases, respectively. The peak at 1596 cm^−1^ originated from the ring mode of adenine or guanine. The peak at 1604 cm^−1^ was believed to originate from the mitochondria, based on an earlier Raman microspectroscopic study by the Hamaguchi group using isolated mitochondria from yeast [59].

### SERS profiles from cardiomyocytes

The SERS profiles from 12-d grown beating cardiomyocyte tissues loaded with 100 nm GNPs were measured using 1-s laser exposures. In the case of ES cells and EBs, 30-s exposures were used. In **Figure 4F**, the peak at 645 cm^−1^ was assigned to the C-C twist of tyrosine, and the peak at 787 cm^−1^ corresponded to the O-P-O stretch band of DNA and RNA [52]. In addition, the peaks at 900 and 949 cm^−1^ were ascribed to the C-C skeletal stretch in proteins and the C-C skeletal random mode in proteins, respectively [51]. The very strong peak at 1006 cm^−1^ originated from the phenylalanine ring vibration mode [51]–[55]. The medium peak at 1190 cm^−1^ was assigned to nucleotide bases as well as to tryptophan and phenylalanine. The medium peaks at ∼1223 cm^−1^ were assigned to amide III (O-sheet) [57], [26]. The strong peak at ∼1400 cm^−1^ was assigned to the COO- symmetric stretching of amino acids [58], [61], and 2 other very strong peaks at 1484 and 1494 cm^−1^ were assigned to the adenine or guanine and adenine or thymine bases, respectively. The very strong peak at 1604 cm^−1^ was regarded as the mitochondrial signature. In addition, the peak at 1647 cm^−1^ was assigned to amide I [19], [53].

### Spatiotemporal SERS measurements from cardiomyocyte tissues

**Figure 5:**
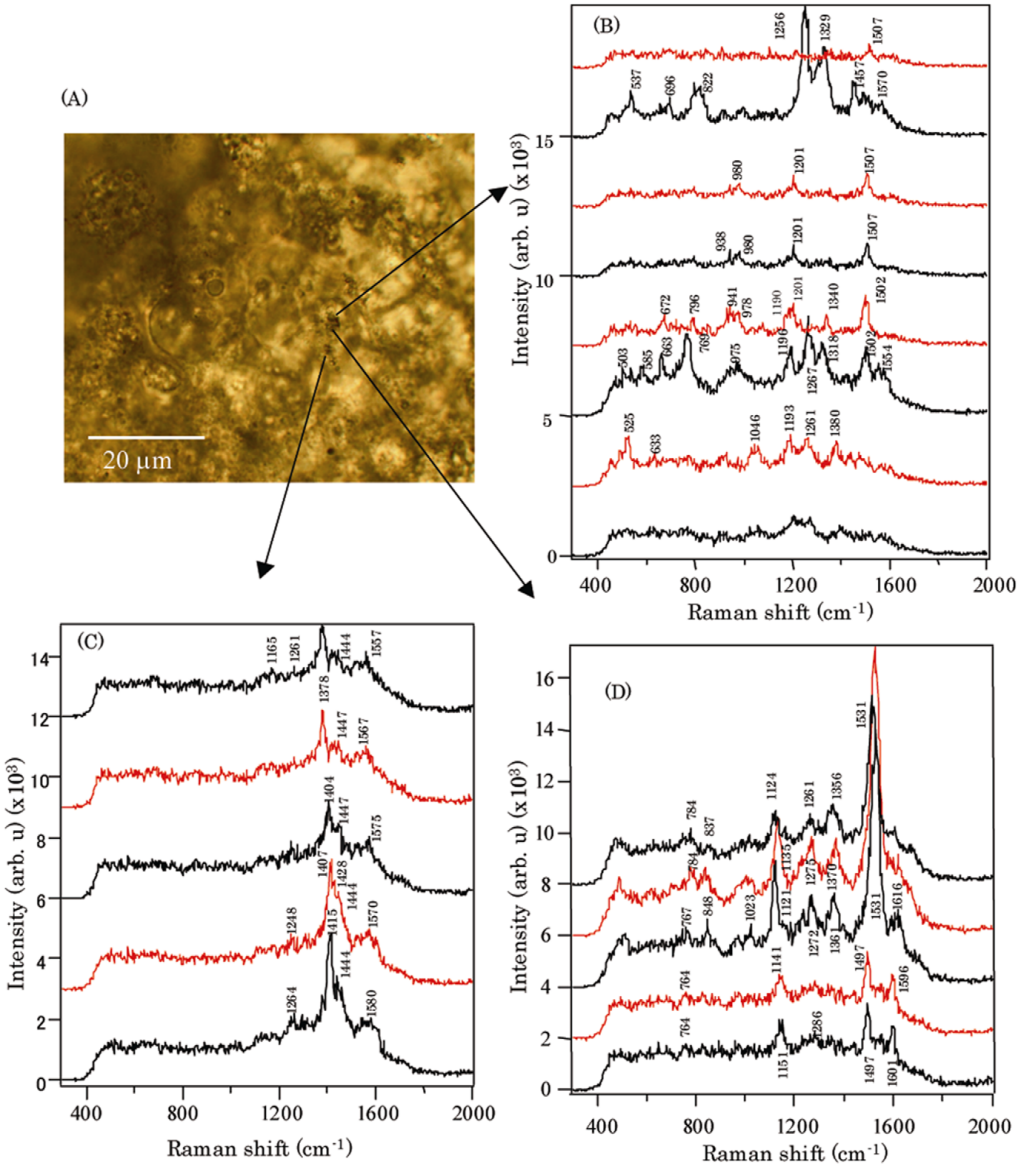
Spatiotemporal measurements of the SERS spectra derived from 100-nm GNP aggregates in 12-d-grown cardiomyocyte tissue in glass-bottom chamber dishes (A). The SERS spectra (B–D) from different GNP aggregates. The spectra were acquired at an exposure of 1 s employing He-Ne (632.8 nm) laser excitations at a power of 130 µW, as shown by the arrows in **Figure 5A**.

**Figure 5A** shows the micrograph of cardiomyocyte tissue. This is the same as the sample shown in **Figure 4C**, and the SERS spectra collected from 3 different locations are shown in **Figures 5B–D**. The SERS spectra, although from the same cardiomyocyte tissue, showed a different peak pattern, which reflects the intracellular biomolecular distribution profile at these locations. The spectral fingerprints further underwent drastic changes for every 1-s exposure, suggesting that temporal changes occur in the chemical nanoenvironment of the GNPs inside the cardiomyocyte. Apart from the changes that occur in the spectral signatures, increases in the SERS signal strength were also noted. Occasionally, a few new peaks appeared besides to the red shift observed in few peaks are also shown in **Figures 5B–D**, which could be attributed to intracellular biomolecular dynamics and transformations. The Raman peaks shown in these spectra are also summarized in Table 1. Briefly, Raman peaks related to amino acids and proteins were predominant in the spectral profile. We also performed time-dependent scans for undifferentiated single ES cells containing 40 nm nanoparticles (**Figure S2**), and EBs containing 60 nm GNP (**Figure S3**) represented more of nucleic acids and bases related peaks in undifferentiated ES cells. We also confirmed the expression of the cardiomyocyte protein α-actinin in 12-d beating cardiomyocyte tissues by carrying out immunostaining experiments (**Figure S4**).

### Identification of Raman peaks related to differentiation

**Figure 6:**
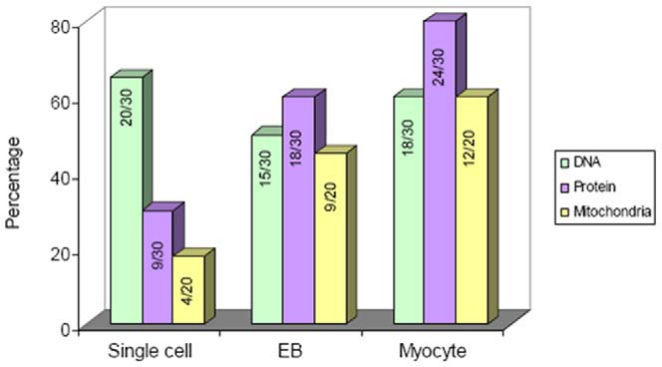
Quantitative SERS profiling of DNA/RNA, protein translation activities (amide I, amide II, amide III), and mitochondrial metabolic activities in single ES cells, EBs, and cardiomyocytes. Calculations are expressed as percentage values. Thirty samples were used to measure the DNA/RNA and protein translation activities, and 20 samples were taken to determine the mitochondrial activities from each stage of ES cell differentiation.

As mentioned above, many of the SERS peaks had overlapping biochemical contributions, which is a common problem in complex biological environments such as those found inside cells. However, the purpose of this study was to identify specific Raman peaks that could serve as probes for ES cell differentiation. Therefore, we adopted a realistic approach by focusing on this aspect of the study and considering cellular molecular function during differentiation. SERS peaks derived from undifferentiated single cells were mostly attributed to nucleic acids (DNA and RNA), as shown in **Figure 4D** and **Figure S2**. This is due to the high proliferation rate of undifferentiated ES cells and is in agreement with reports from mouse and human ES cells based on Raman microspectroscopy [54], [56]. On the other hand, it is noteworthy that the Raman peak at 1604 cm^−1^ that originated from the mitochondria was specifically observed in EBs and cardiomyocytes. This is strongly supported by the ultramicrograph data (**Figures 3A–C**) and reflects the high metabolic activity of EBs and cardiomyocytes. In comparison to undifferentiated single ES cells (**Figure 4A** **and Figure S2**), many Raman peaks related to proteins, such as amide I, amide II, amide III, COO^−^ symmetric stretching, and CH_3_ deformation, were observed in the region 1200–1700 cm^−1^ in EBs and cardiomyocytes (**Figures 4B, 4C** **, and** **5** **; Figure S3**). This suggested that cell activity was at its maximum and driving the cell toward its cellular fate. Therefore, terminally differentiated cardiomyocytes carried out protein translation, post translation, and cell signaling activities unlike undifferentiated single ES cells during differentiation. In addition, SERS analysis clearly demonstrated that EBs are not simple aggregates of ES cells but are an early stage of embryonic differentiation in which protein translation occurs at almost the same level as in differentiated cardiomyocytes. Furthermore, we quantitatively calculated 3 major molecular signatures—those of DNA/RNA, protein translation activities (amide I, amide II, and amide III), and mitochondria. These represent the high metabolic activities of cells during the 3 stages of ES cell differentiation, i.e., undifferentiated single cells, EBs, and differentiated cardiomyocytes (shown in **Figure 6**). As expected, undifferentiated single-cell derived SERS spectra exhibited abundant DNA-RNA-related peaks (O-P-O stretch DNA: 787 cm^−1^ and O-P-O stretch RNA: 813 cm^−1^). Moreover, EBs and terminally differentiated cardiomyocytes showed SERS spectra predominantly from proteins (amide I, amide II, and amide III) and mitochondria (1604 cm^−1^); these are expressed as percentage values.

Thus, we demonstrated that the SERS-based analysis of ES cells has great potential in identifying Raman spectral features specific to each stage of differentiation. Conventional Raman scattering analysis of biological cells yields ensemble-averaged information of numerous different molecules. In contrast, SERS arises from a limited number of molecules adsorbed onto metal nanoparticles and captures detailed molecular information that is buried in ensemble-averaged conventional Raman spectra. This study demonstrated that GNP uptake did not inhibit cell viability but instead supported the proliferation of mouse ES cells. GNP internalization was mostly localized to the mitochondria in all 3 differentiation stages of mES cells and was assumed to be cell-specific. The results indicated that this method could be used to deliver drugs or probes to mitochondria without compromising the cell viability for *in situ* and real-time imaging and monitoring of mitochondrial molecular dynamics. GNP administration further enabled fast and noninvasive SERS-based molecular profiling of ES cell differentiation. The results proved the advantages of this method over other biochemical methodologies currently in use for the quick profiling of mES cells without the use of any labels in small numbers or even at the single cell level. Further extension of the SERS-based method used in this study to induced pluripotent stem (iPS) cells would probably reveal the molecular and biochemical differences that exist between ES and iPS cells. Such studies could also contribute to our understanding of the reprogramming of somatic cells to iPS cells or specialized cells.

## Supporting Information

Figure S1 Energy dispersive X-ray (EDX) spectrum analysis of GNPs accumulated cardiomyocyte tissues by 200 kV EMAX7000 (Horiba, Japan) coupled with HRTEM (Hitachi, H-8000). Peaks at 2.12 and 9.71 keV energy levels unique to gold element, while “Cu” peak comes from copper grid that employed for TEM specimen and “U” peak stands for uranyl acetate staining of tissue specimen for better visualization of intracellular organizations.
(TIF)

Figure S2 Time-dependent measurement of SERS spectra from single ES cell treated with 40 nm GNPs. Each spectrum acquired with 30 s He-Ne laser exposures. Spectra were measured from the particle indicated in the circle.
(TIF)

Figure S3 Time-dependent measurement of SERS spectra from single EB treated with 60 nm GNPs. Each spectrum acquired with 30 s He-Ne laser exposures. Spectra were measured from the aggregate indicated in the circle.
(TIF)

Figure S4 Immunostaining of beating cardiomyocyte tissue derived from 12-d grown EB loaded with gold nanoparticles (100 nm) expressing α-actinin, myofibriallar protein specific to cardiomyocytes. GFP (A), TRITC-labeled α-actinin (B) DAPI (C), and overlap (D). Scale bar 100 µm. In brief, 12-d grown cardiomyocyte tissues were fixed with 4% (v/v) paraformaldehyde for 20 min at RT followed by treatment with 2% (v/v) Triton X-100 and blocking was achieved by 3% (w/v) BSA dissolved in PBS buffer for 1–2 h at RT and incubated the specimens with a cardiac specific primary antibody α-actinin (500 times dilutions) overnight at 4°C. Specimens were washed with 0.05% Tween-20 in PBS and then tissue specimens were incubated with TRITC-labeled secondary antibody (SC 2092, 200 times dilution) for 3 h at RT. DAPI was used for staining the nucleus followed by PBS washings and specimens were mounted in 2–3 drops mounting solution and examined microscopically.
(TIF)
