# Supplementary figures and images for: Gold Nanoparticle-Based Surface-Enhanced Raman Scattering for Noninvasive Molecular Probing of Embryonic Stem Cell Differentiation
Source: PLoS One. 2011 Aug 4;6(8):e22802. doi: 10.1371/journal.pone.0022802 (PMC3150363; doi:10.1371/journal.pone.0022802)

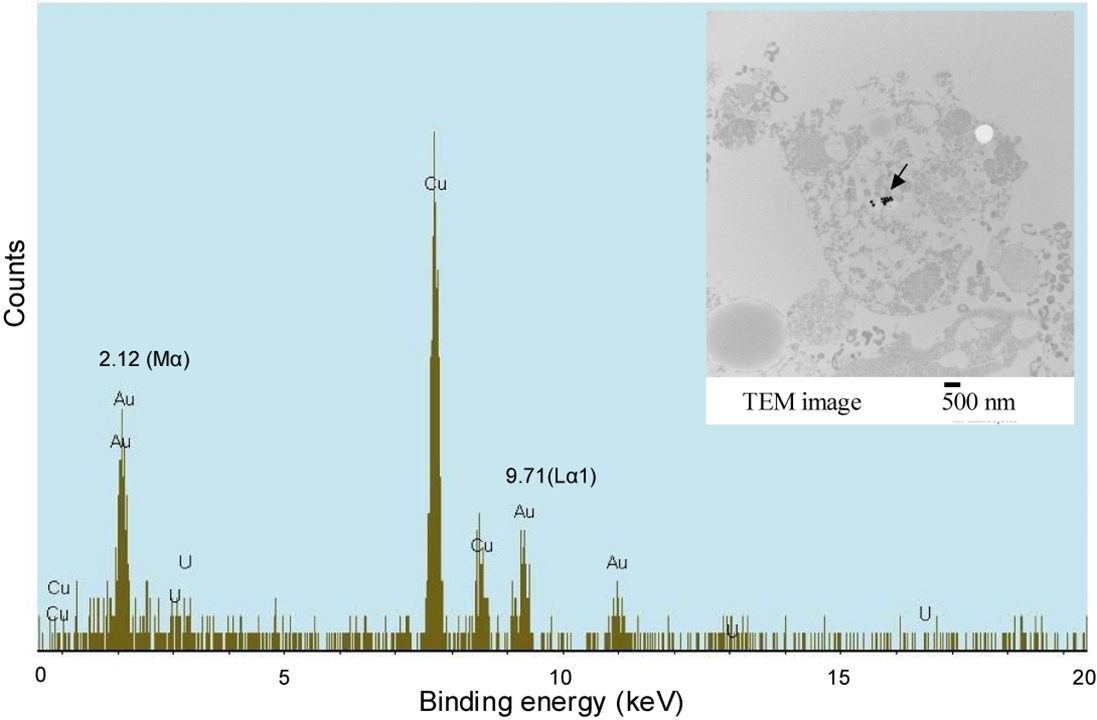

Supplement: Figure S1 — Energy dispersive X-ray (EDX) spectrum analysis of GNPs accumulated cardiomyocyte tissues by 200 kV EMAX7000 (Horiba, Japan) coupled with HRTEM (Hitachi, H-8000). Peaks at 2.12 and 9.71 keV energy levels unique to gold element, while “Cu” peak comes from copper grid that employed for TEM specimen and “U” peak stands for uranyl acetate staining of tissue specimen for better visualization of intracellular organizations. (TIF) [file pone.0022802.s001.tif]

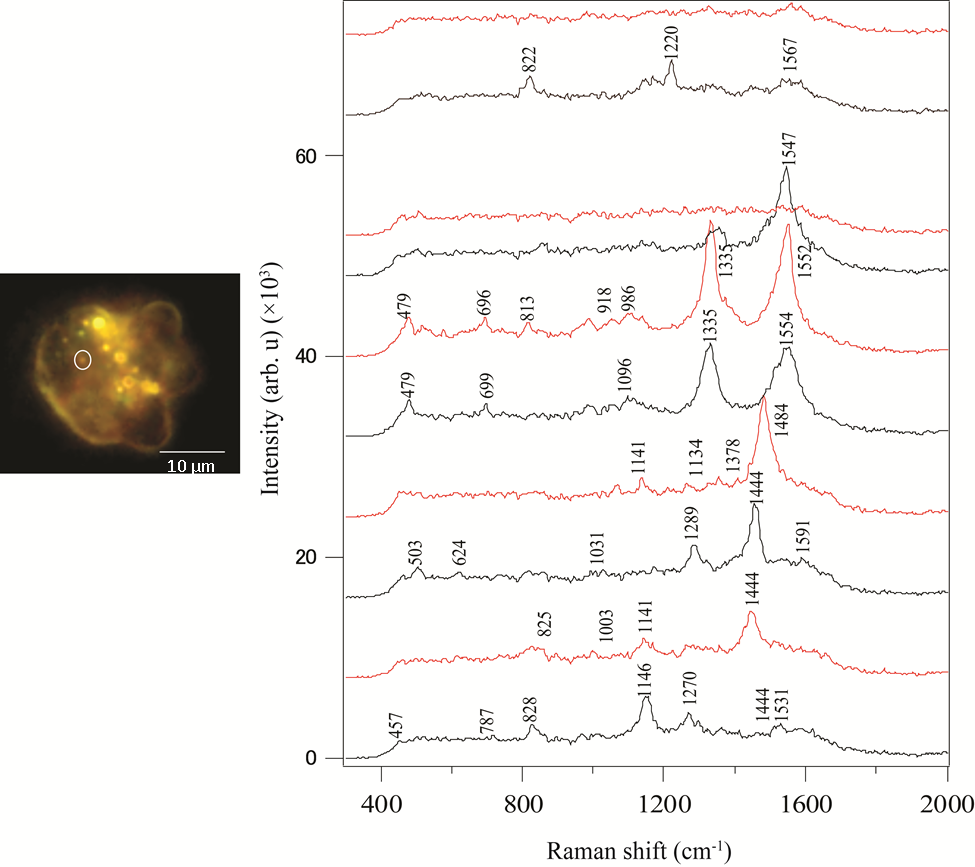

Supplement: Figure S2 — Time-dependent measurement of SERS spectra from single ES cell treated with 40 nm GNPs. Each spectrum acquired with 30 s He-Ne laser exposures. Spectra were measured from the particle indicated in the circle. (TIF) [file pone.0022802.s002.tif]

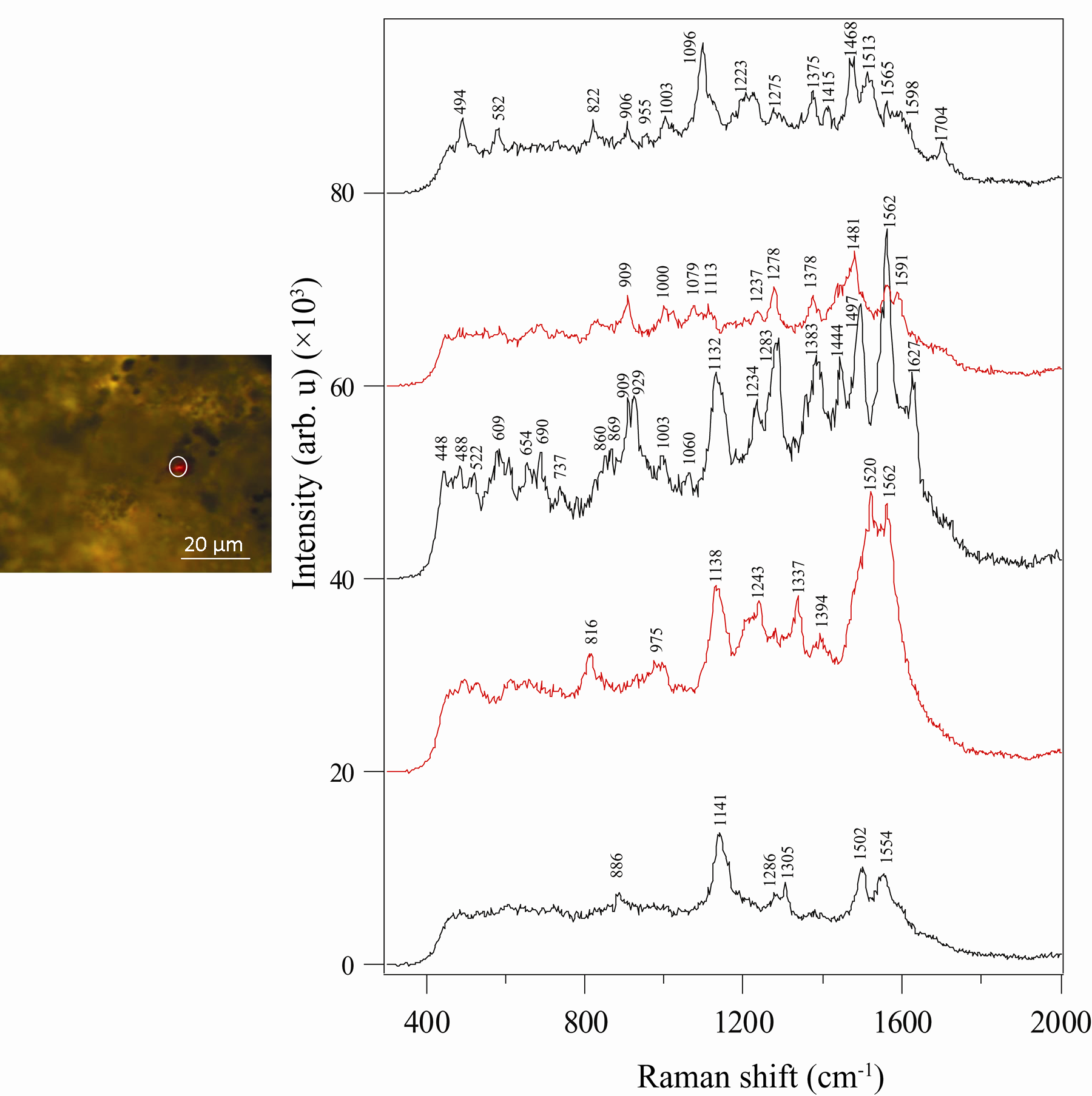

Supplement: Figure S3 — Time-dependent measurement of SERS spectra from single EB treated with 60 nm GNPs. Each spectrum acquired with 30 s He-Ne laser exposures. Spectra were measured from the aggregate indicated in the circle. (TIF) [file pone.0022802.s003.tif]

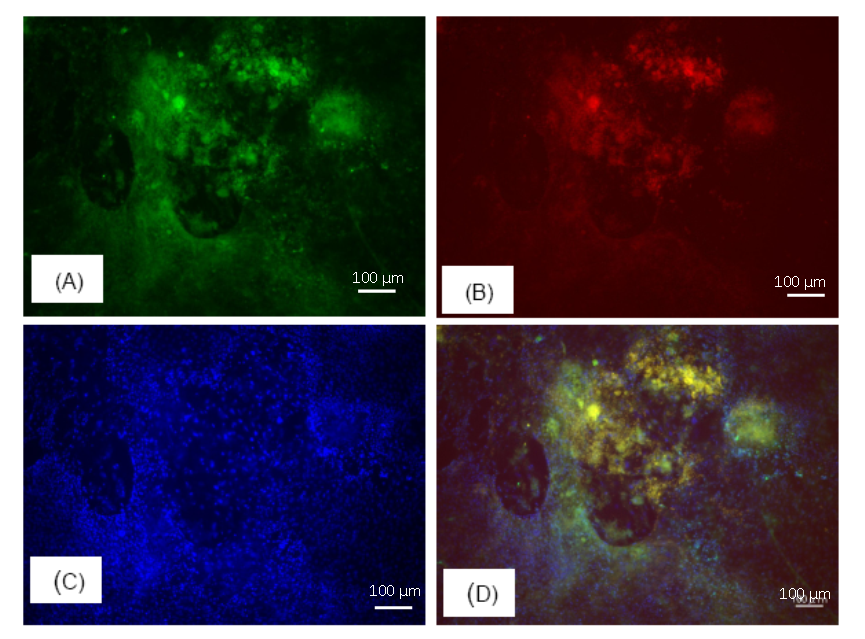

Supplement: Figure S4 — Immunostaining of beating cardiomyocyte tissue derived from 12-d grown EB loaded with gold nanoparticles (100 nm) expressing α-actinin, myofibriallar protein specific to cardiomyocytes. GFP (A), TRITC-labeled α-actinin (B) DAPI (C), and overlap (D). Scale bar 100 µm. In brief, 12-d grown cardiomyocyte tissues were fixed with 4% (v/v) paraformaldehyde for 20 min at RT followed by treatment with 2% (v/v) Triton X-100 and blocking was achieved by 3% (w/v) BSA dissolved in PBS buffer for 1–2 h at RT and incubated the specimens with a cardiac specific primary antibody α-actinin (500 times dilutions) overnight at 4°C. Specimens were washed with 0.05% Tween-20 in PBS and then tissue specimens were incubated with TRITC-labeled secondary antibody (SC 2092, 200 times dilution) for 3 h at RT. DAPI was used for staining the nucleus followed by PBS washings and specimens were mounted in 2–3 drops mounting solution and examined microscopically. (TIF) [file pone.0022802.s004.tif]
